# Pre-transplant Loco-Regional Therapy for Hepatocellular Carcinoma and Post-transplant Outcomes: A National Study

**DOI:** 10.7759/cureus.67960

**Published:** 2024-08-27

**Authors:** Jay Desai, Raymond I Okeke, Roshani Desai, Zidong Zhang, Annabel Engelhardt, Mark Schnitzler, John Barron, Chintalapati R Varma, Henry B Randall, Krista L Lentine, Mustafa Nazzal

**Affiliations:** 1 Department of Surgery, Saint Louis University Hospital, Saint Louis, USA; 2 Gastroenterology, Saint Louis University School of Medicine, Saint Louis, USA; 3 Surgery, Saint Louis University School of Medicine, Saint Louis, USA; 4 Center for Abdominal Transplantation, Saint Louis University School of Medicine, Saint Louis, USA

**Keywords:** hepatic tumors, tumor, solid organ transplant, loco-regional therapy, hepatocellular carcinoma

## Abstract

The ultimate preferred treatment for hepatocellular carcinoma (HCC) complicated with cirrhosis and portal hypertension is an orthotopic liver transplant (OLT). Loco regional therapy (LRT) has emerged to prevent tumor growth and progression of disease beyond the Milan criteria to achieve transplant. There is a paucity of data regarding safety, posttransplant survival benefits, and tumor recurrence rate achieved by these LRT modalities. We aim to assess and compare the five-year survival rate and tumor recurrence rate with or without LRT in patients after OLT with diagnosed HCC utilizing the nation's largest dataset. This is a retrospective observational study approved by Saint Louis University institutional review board. We utilized the largest dataset from the years 2003-2013 where pertaining data were gathered from Organ Procurement Transplant Network (OPTN) standard analysis and research files (STAR) through novel linkages with Medicare bills. Descriptive and comparative statistics were performed. 2412 (51.6%) patients received any form of locoregional therapy (single or combination) out of 4669 total study sample size. The overall five-year survival in the study sample was 76.1%. There was statistically no significant improvement seen in five-year posttransplant survival in the group that received one mode of LRT (adjusted hazard ratio (aHR) 0.97, P<0.64) or a combination of LRT (aHR 0.94, P<0.58) in comparison to those that received none after adjusting donor and recipient clinical characteristics. However, five-year survival trended higher among those treated with combination therapy over those treated with single LRT or none. Overall HCC recurrence was 4.8%, while no significant difference was noted when comparing above-mentioned groups. Five-year posttransplant survival and HCC recurrence rate were also found to have no difference when compared between above-mentioned groups after adjusting explant pathology.

This is the largest retrospective study comparing liver transplant patients with HCC who received LRT to none. Although it did not show any statistically significant benefit of single or combination of LRT on survival or tumor recurrence after liver transplant for HCC patients, the outcomes encourage the safe and feasible use of LRT as a bridging therapy. Our study also suggests an observed pattern of improved posttransplant survival and tumor recurrence rate with combination loco-regional therapy. Larger multicenter prospective studies will be required to achieve the effect size to determine the best therapies for maximizing patient survival cost-effectively.

## Introduction

Orthotopic liver transplant (OLT) is the ultimate treatment for early-stage hepatocellular carcinoma (HCC) established from the milestone study by Mazzaferro in 1996, as it simultaneously treats the tumor and underlying liver cirrhosis and portal hypertension [1,2]. Liver organ allocation system was revolutionized in February 2002 after implementation of the model for end-stage liver disease (MELD) scoring system that could accurately predict three-month mortality in liver waitlist patients [3]. However, this scoring system had limitations in explaining the severity of illness in certain patients including HCC pertinent to tumor expansion, which prompted the implementation of a standardized system of exception points from 2003 onwards [4]. Since then, several changes have been made to the Organ Procurement Transplant Network (OPTN) regarding HCC MELD exception points after they qualify for Milan Criteria, leading to an anticipated six-month waiting period before exception points are granted [4]. Increase in wait time led to progression of disease beyond Milan criteria, making patients ineligible for transplant for which the drop-out rate was reported as high as 30-40% in the literature [5]. Loco-regional therapy (LRT) emerged and has been used for years to control tumor growth and prevent progression beyond Milan criteria and occasionally to downstage patients and bridge them over time until they are eligible for transplant.

To reduce the drop-out rate, transplant centers have initiated the use of bridging LRT early in the presentation of HCC, specifically transarterial chemoembolization (TACE), radiofrequency ablation (RFA), radioembolization y90 therapy (RT), and combinations of these three therapies. The safety and feasibility of these therapies have been demonstrated in the literature [6,7]. Although prevention of drop-out rate with LRT seems intuitive, there is a paucity of data regarding the posttransplant survival benefit, or the disease-free interval achieved by these modalities. Moreover, which LRT modality or combination modalities seem to be more successful is another question that needs an answer. Even after having a successful OLT, another important factor to consider is the pathology and other micro characteristics of the patient's HCC explant pathology, as these can affect long-term survival and recurrence. While there are several studies investigating LRT as a bridging therapy for patients with HCC, data on this topic is not comprehensive or complete. These studies overall have not shown a significant increase in survival rate or reduction in posttransplant recurrence rate [8-13]. Furthermore, most of these studies do not use explant pathology for risk adjustment to account for patients who underwent transplant, with subsequent explant pathology revealing HCC with a higher risk for recurrence (i.e., increase in tumor size in comparison to pre-transplant imaging, poorly differentiated tumors, or an increased number of lesions outside of the Milan criteria).

We aimed to utilize a larger dataset to assess the primary outcomes of five-year survival rate and tumor recurrence rate after orthotopic liver transplant in patients with diagnosed HCC who received and did not receive preoperative bridging LRT. The primary outcomes were also assessed among single vs combined LRT groups after adjustment for pre-transplant recipient and donor characteristics and after adjustment for explant pathology. We also examined the MELD score changes with LRT to assess the safety of LRT before transplantation.

## Materials and methods

Data abstraction

Data was collected from the OPTN system. This data included donors, wait-listed candidates, and transplant recipients in the U.S., as submitted by the members of OPTN. The Health Resources and Services Administration (HRSA), an agency of the U.S. Department of Health and Human Services, provides oversight of the activities of the OPTN contractor. We utilized Medicare coverage patient population for inclusion in study sample as the data pertinent to pre-transplant LRT and their type would be available for study objective. Clinical, demographic, and claims variables for adult patients (age >18) who received liver transplantation, with HCC as the primary diagnosis for liver failure, between 2003-2013 were obtained from a database linking OPTN Standard Analysis and Research (STAR) files with Medicare billing claims. Medicare billing claims included diagnostic and procedure codes for patients with Medicare fee-for-service primary or secondary insurance.

Data regulation

After regulatory approval, beneficiary identifier numbers from Medicare's electronic databases were linked to Social Security Numbers, gender, and birthdates to unique OPTN identifiers. Analyses were performed in compliance with the Health Information Portability and Accountability Act (HIPAA); all direct identifiers were removed from limited datasets. Because of the large sample size, the anonymity of the patients studied, and the non-intrusive nature of the research, a waiver of informed consent was granted per the Department of Health and Human Services Code of Federal Regulations (Title 45, Part 46, Paragraph 46.116). Analyses were performed using HIPPA-compliant limited datasets. This study was reviewed and approved by the Saint Louis University Institutional Review Board.  

Locoregional therapy

Patients who received LRT were identified based on the International Classification of Disease (ICD)-9 procedures and Common Procedural Terminology (CPT) codes for treatments undergone within the one-year preceding OLT. Based on study data findings, LRT was then further characterized into five groups: TACE, RFA, trans-arterial RT, alcohol injection (AI), or any combination of therapies listed above. In addition, clinical and demographic characteristics of the recipient, characteristics of the donated organ, and other transplant factors were outlined by the OPTN Transplant Candidate and Recipient Registration forms (Appendix 1). With this information, the pre-transplant HCC tumor characteristics were closely examined (Appendix 2), since HCC pathology is known to influence post-OLT recurrence and death. The OPTN began collecting this specific explant pathology information in April 2012; because this information was not available previously, we were only able to examine the explant pathology data reported to the OPTN after April 2012 (Appendix 3).

Primary outcomes

Our primary outcomes were patient five-year Survival and HCC recurrence. The five-year posttransplant survival and HCC recurrence rates were identified by OPTN reports. To obtain five-year posttransplant survival rate we utilized crude mortality rate from any cause, reported them in percentage of study sample size and subtracted from 100. HCC recurrence was defined as the earliest reported date of posttransplant diagnosis of HCC or HCC as reported cause of death.

Secondary subgroup analysis

For comparison of primary outcomes among LRT subgroups, multivariate analysis was carried out by Cox-regression adjusted for donor and recipient demographic and clinical characteristics. Donor characteristics utilized for analysis were donor age, race, donor diabetes status, donor risk index (DRI), donor share type, donor types (such as living or deceased donor), and cold ischemia time. Recipient characteristics were age, race, gender, BMI, co-morbidities, education status, employment status, MELD score, blood type etc. Multivariate analysis was also performed by adjusting for explant pathology that includes tumor size (<=8 cm or >8 cm), number of lesions, presence of vascular invasion, and differentiation as reported to the OPTN (Appendices 1, 3).

MELD score

The MELD score was calculated at the time of transplantation and then compared with the listed MELD score. The listing MELD score gives the closest approximation of the MELD score before any LRT. The change in MELD score was utilized to estimate LRT treatment safety as it has been reported to cause liver decompensation and increase MELD score due to adverse effects.

Statistical analyses

Data management and analysis were performed using SAS 9.4 for Windows (SAS Institute Inc., Cary, NC, USA). Patient baseline characteristics were compared among LRT regimens using the Chi-square test for categorical variables and ANOVA for continuous variables. Kaplan-Meier survival analysis was used to assess patient posttransplant survival of different treatment regimens compared to no treatment for up to five years posttransplant. Multivariate Cox regression was conducted to adjust for baseline recipient and donor transplant factors, as well as explant pathology, including tumor size, number of tumors, presence of vascular invasion, and tumor differentiation as reported to the OPTN.

**Figure 1:**
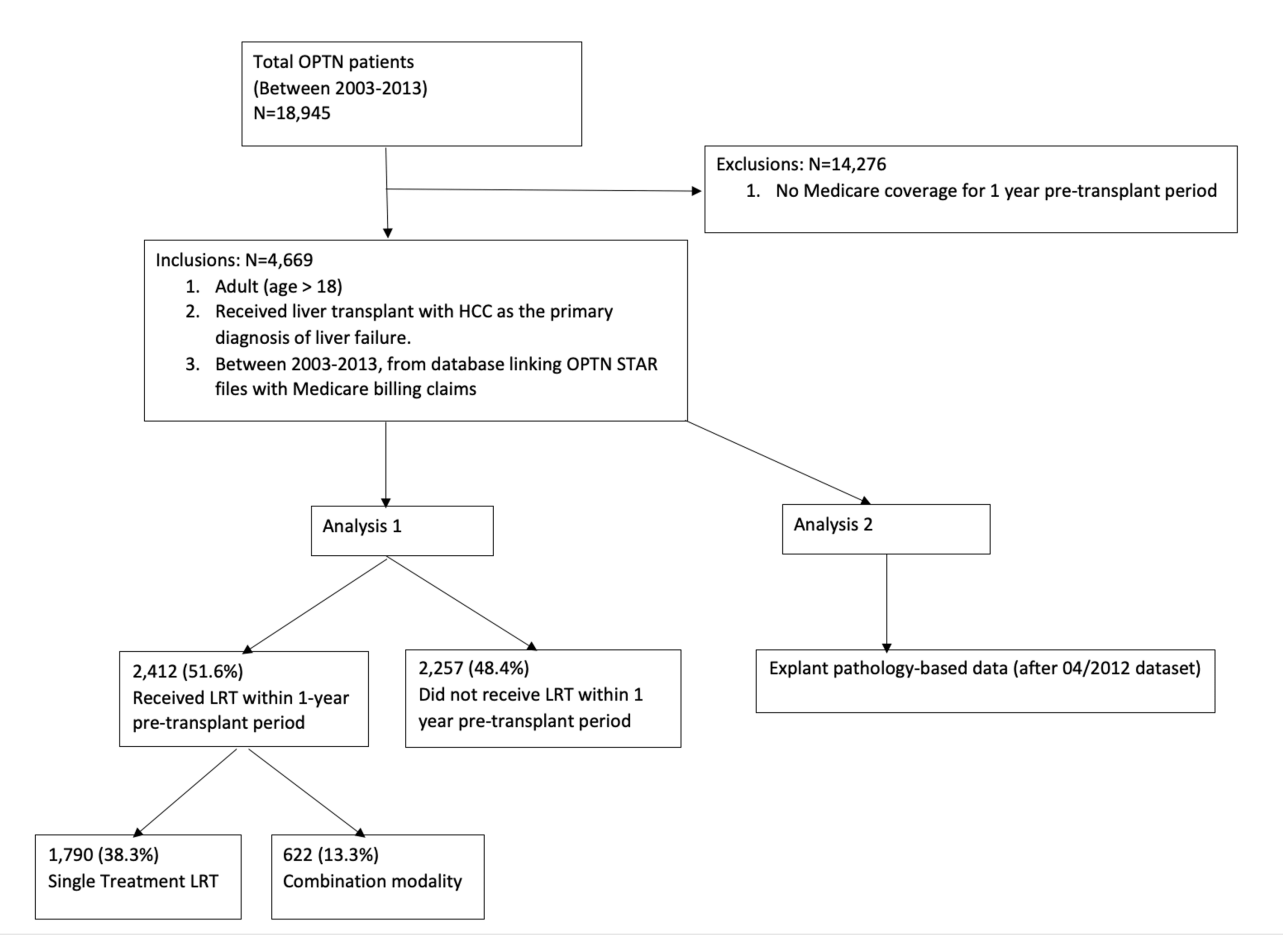
Methods OPTN - Organ Procurement and Transplantation Network; HCC - Hepatocellular carcinoma; STAR - Standard Transplant Analysis and Research; LRT - Locoregional therapy

See Figure 1 below for methods breakdown.

## Results

From the total U.S. transplant recipients in the study period with a primary diagnosis of HCC (n=18,945), 4,669 was the actual sample size based on the study inclusion criteria of data availability with Medicare coverage for one year before transplant. Out of the total sample size, 2,412 patients received LRT within one year before transplantation, while 2,257 patients did not receive LRT (Table 1).

**Table 1:** Summary of treatment distribution for eligible patients (n = 4,669) *Highlighted are the groups included in the multivariate analysis.  LRT - Locoregional therapy

PRIOR LRT TREATMENT	n	Percent of Total Eligible Patients
NO LRT TREATMENT*	2,257	48.3
WITH LRT TREATMENT*	2,412	51.6
SINGLE LRT MODALITY	1,790	38.3
COMBINATION LRT MODALITIES	622	13.3

The treatment group was then subdivided into single LRT therapy (one treatment modality), including TACE only (n=1,399), RFA only (n=390), alcohol injection only (n=1), or combination therapy of two or more LRT modalities (n=622). A minority of patients were managed with Y90 therapy (RT) in combination with one other treatment modality (n=81) (Table 2). Recipient, donor, and tumor characteristics were generally similar when comparing the different treatment groups. The overall five-year crude mortality in the study sample was 23.9% (Table 3).

**Table 2:** Breakdown of treatment options for treated patients (n = 2,412) *Highlighted are the groups included in the multivariate analysis.  LRT - Locoregional therapy; TACE - trans-arterial chemoembolization; RFA - radiofrequency ablation; RT - Radio-embolization y90 therapy; AI - alcohol injection

LRT TREATED PATIENTS	n	Percent of Total Treated Patients
TOTAL TREATED PATIENTS	2,412	100
SINGLE TREATMENT	1,790	74.2
TACE ONLY	1,399	58.0
RFA ONLY	390	16.2
RT ONLY	0	0.0
AI ONLY	1	0.0
COMBINATION TREATMENT	622	25.8
TACE+RFA	352	14.6
TACE+RT	65	2.7
TACE+AI	156	6.5
RFA+RT	0	0.0
RFA+AI	1	0.0
RT+AI	0	0.0
TACE+RFA+RT	15	0.6
TACE+RFA+AI	32	1.3
RFA+RT+AI	0	0.0
TACE+RFA+RT+AI	1	0.0

**Table 3:** Overall frequency of mortality at 5 years posttransplant

Survival	3,553	76.1%
Mortality	1,116	23.9%

**Figure 2:**
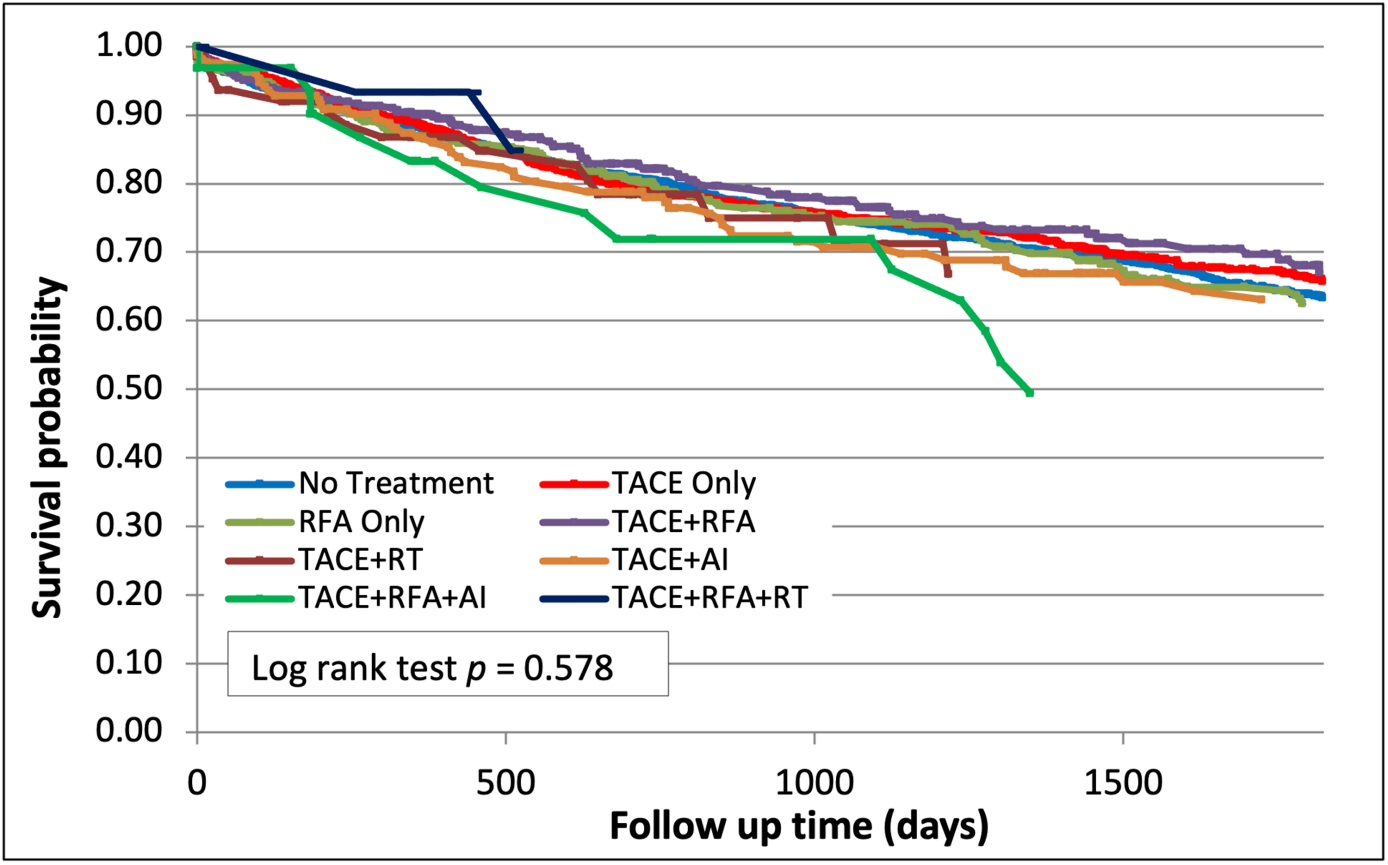
Survival after LRT (p=0.58) LRT - Locoregional therapy; TACE - trans-arterial chemoembolization; RFA - radiofrequency ablation; RT - Radio-embolization y90 therapy; AI - alcohol injection

Five-year posttransplant survival of the study sample was found to be 76%. In this, the group that was not treated with LRT had a survival of 63.4% (p=0.58, Figure 2). Five-year posttransplant survival among those treated with TACE + RFA was 67.1%, TACE + RT was 66.8%, and TACE + RFA + RT was 84.8% (Figure 2). There was a trend towards improved five-year posttransplant survival in groups that received two or more LRT modalities. However, there was no statistically significant improvement in posttransplant survival between those who received two or more LRT modalities compared to the group that was not treated with LRT (63.4%, p=0.58, Figure 2). 

**Figure 3:**
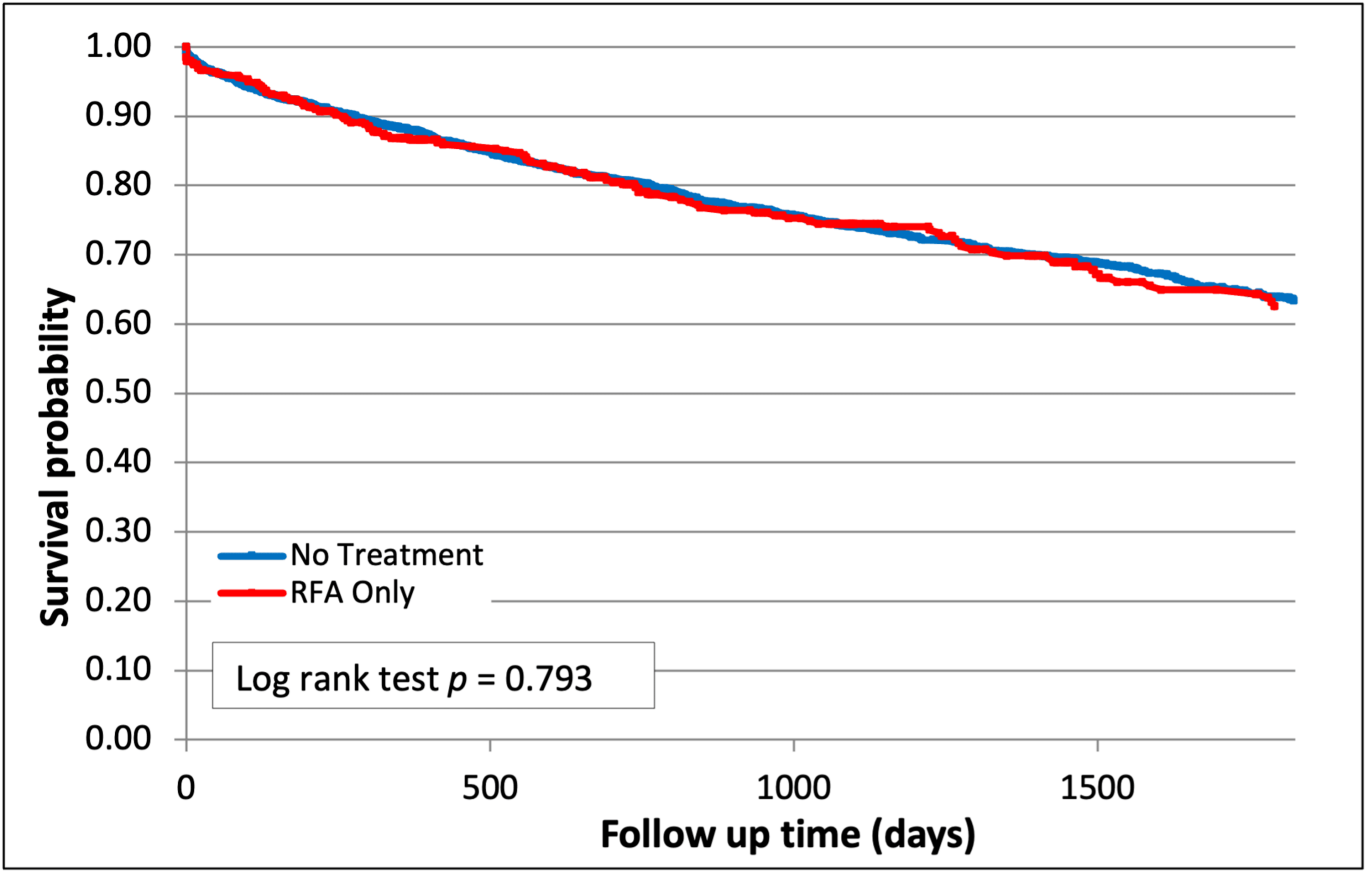
Survival after RFA Therapy (p=0.79) RFA - radiofrequency ablation

Single therapy with RFA alone resulted in no survival difference when compared to no treatment (62% vs 63.4%, p=0.79, Figure 3). 

**Figure 4:**
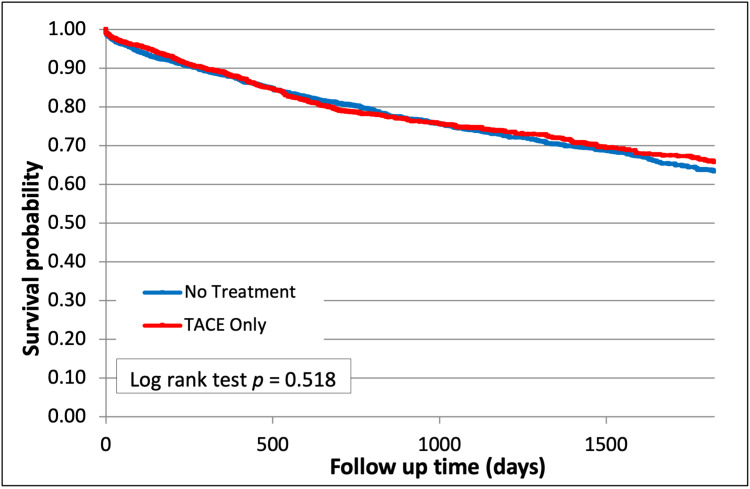
Survival after TACE Therapy (p=0.52) TACE - trans-arterial chemoembolization

Post-transplant survival was also similar among patients who received TACE therapy alone compared with the group that received no treatment (65.8% vs 63.4%, p=0.52, Figure 4). 

**Figure 5:**
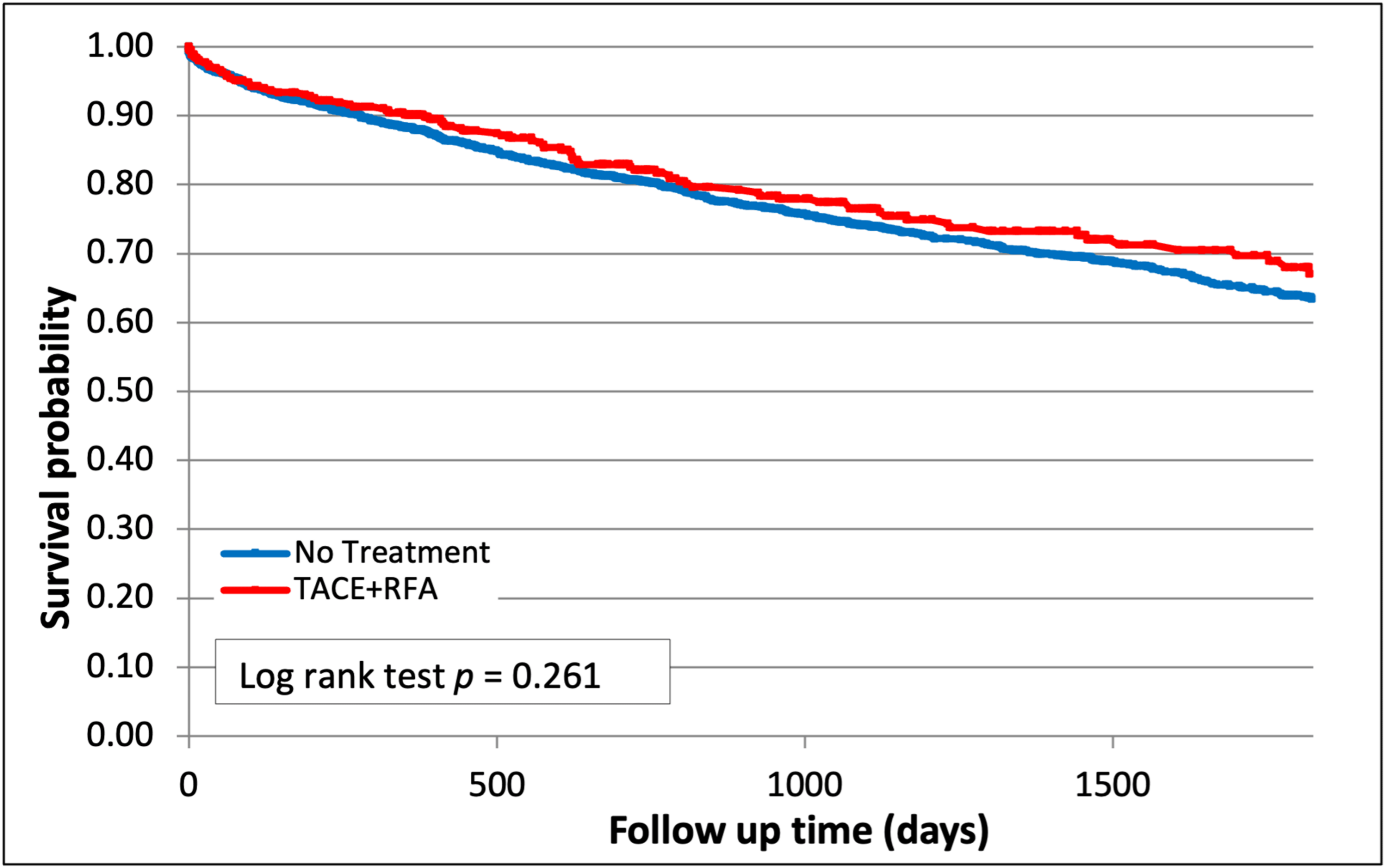
Survival after Combination therapy with TACE and RFA (p=0.26) TACE - trans-arterial chemoembolization; RFA - radiofrequency ablation

In contrast, combination treatment with TACE + RFA showed a trend towards improved survival compared with no treatment (67.1% vs 63.3%, p=0.26, Figure 5) but did not reach statistical significance.

Multivariate adjustment for baseline patient demographic and clinical characteristics and pre-transplant tumor characteristics as reported on imaging, revealed that single therapy (adjusted hazard ratio (aHR) 0.97; 95% CI (0.85, 1.11)) and combination therapy (aHR 0.95; 95% CI (0.79, 1.14)) were associated with no survival benefit or significant improved freedom from recurrence when compared to those that received no LRT (Tables 4, 5). Overall, posttransplant HCC recurrence was identified in 4.8% of recipients (Table 6). 

**Table 4:** Comparison of five-year mortality between treatment and non-treatment groups after adjusting donor and recipient demographics and clinical characteristics. aHR - Adjusted Hazard Ratio

Treatment Group	n	aHR (95% CI)	p-value
No Treatment	2,257	1.00	Reference
Single Treatment	1,790	0.97 (0.85, 1.11)	0.649
Combination Treatment	622	0.95 (0.79, 1.14)	0.589

**Table 5:** Comparison of tumor recurrence rate between treatment and non-treatment groups after adjusting donor and recipient demographics and clinical characteristics. aHR - Adjusted Hazard Ratio

Treatment Group	n	aHR (95% CI)	p-value
No Treatment	2,257	1.00	Reference
Single Treatment	1,790	1.11 (0.83, 1.49)	0.464
Combination Treatment	622	0.93 (0.60, 1.41)	0.719

**Table 6:** Overall frequency of hepatocellular carcinoma (HCC) recurrence at five years posttransplant

No recurrence	4,446	95.2%
Recurrence	223	4.8%

**Figure 6:**
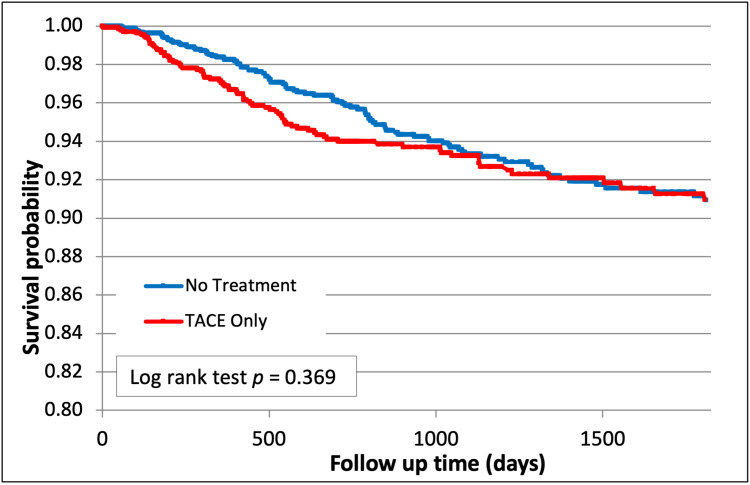
Hepatocellular carcinoma (HCC) recurrence after TACE (p=0.37) TACE - trans-arterial chemoembolization

TACE was associated with a non-significant trend towards improved freedom from recurrence compared to no treatment (91.0% vs 90.0%, p=0.37, Figure 6).

**Figure 7:**
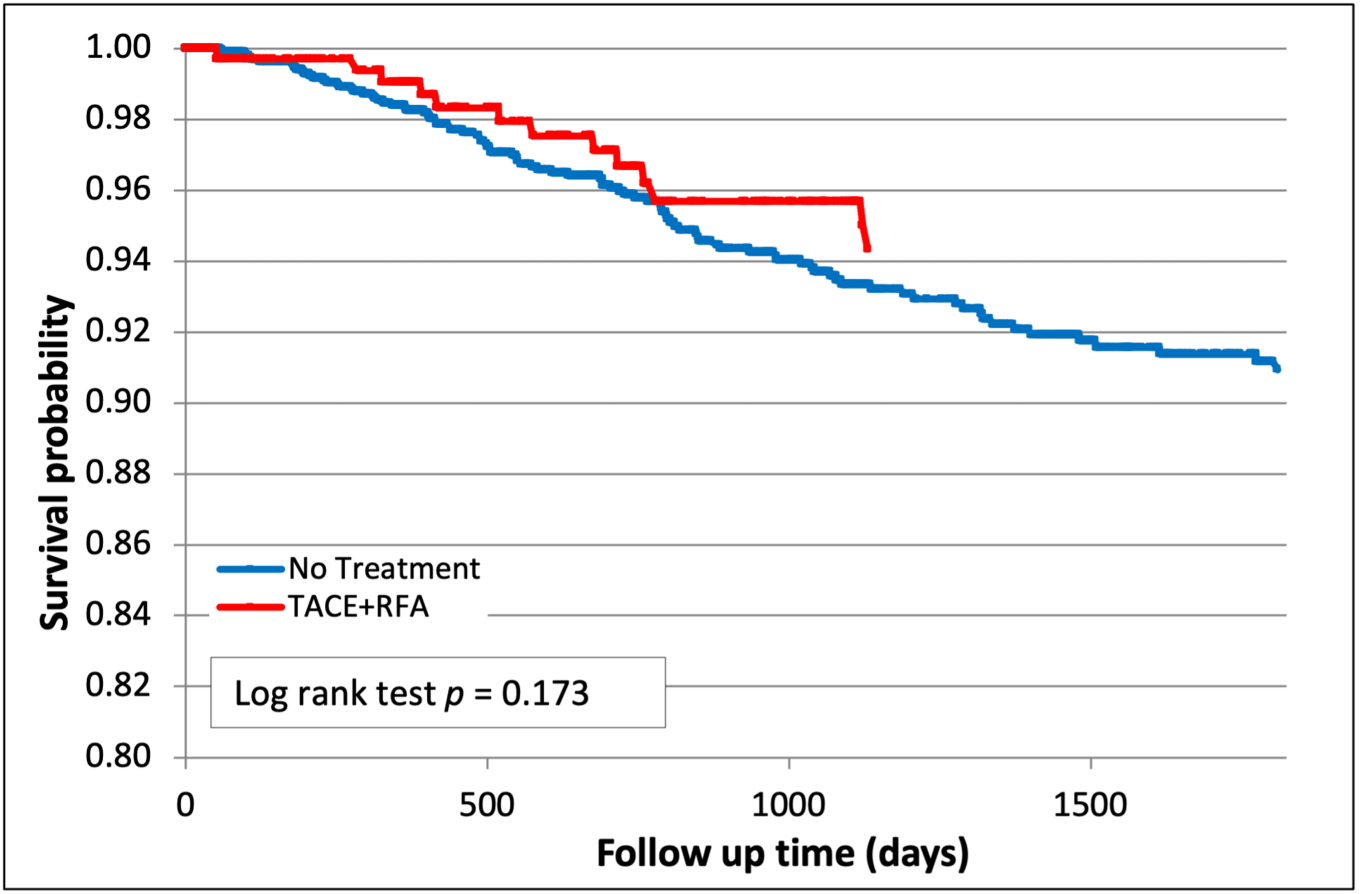
Hepatocellular carcinoma (HCC) recurrence after TACE + RFA (p=0.17) TACE - trans-arterial chemoembolization; RFA - radiofrequency ablation

Combination therapy demonstrated a trend towards improved freedom from recurrence compared to other groups but was not statistically significant. TACE + RFA also showed a non-significant trend towards improved freedom from recurrence compared to no treatment, particularly earlier on (94.3% vs 90.0%, p=0.17, Figure 7). 

Explant pathology

A secondary analysis was performed using tumor explant pathology variables in 1,674 patients (Appendix 3). Single treatment and combination treatment did not have any survival benefit or significant improved freedom from recurrence when compared to no treatment (Tables 7, 8). We noticed a trend towards improved survival with a combination treatment when compared to no treatment that did not reach statistical significance (HR 0.95, p=0.613, Table 7).

**Table 7:** Comparison of five-year mortality between treatment and non-treatment groups after adjusting explant pathology (largest tumor size) obtained from pretransplant imaging. aHR - Adjusted Hazard Ratio

Treatment Group	n	aHR (95% CI)	p-value
No Treatment	2,257	1.00	Reference
Single Treatment	1,790	0.96 (0.84, 1.09)	0.509
Combination Treatment	622	0.95 (0.80, 1.14)	0.613

**Table 8:** Comparison of tumor recurrence rate between treatment and non-treatment groups after adjusting explant pathology (largest tumor size) obtained from pretransplant imaging. aHR - Adjusted Hazard Ratio

Treatment Group	n	aHR (95% CI)	p-value
No Treatment	2,257	1.00	Reference
Single Treatment	1,790	1.09 (0.81, 1.45)	0.568
Combination Treatment	622	0.92 (0.60, 1.41)	0.701

MELD score before and after LRT

MELD scores were reviewed at the time of listing for transplant and then again at the time of transplant to determine if LRT, (especially combined treatment modalities) is safe for patients, and does not worsen liver function, as reflected by an increase in the MELD score after receiving LRT. No significant change in MELD scores was identified between those who received no LRT, those who received single modality therapy, and those who received combined therapies (Table 9).

**Table 9:** Model for end-stage liver disease (MELD) score before and after loco-regional therapy (LRT)

Treatment Group	MELD At Addition to Waitlist	MELD At Transplant	MELD Score Change
No treatment	13.8 (6.8)	11.6 (4.3)	2.1 (5.2)
Single Treatment	13.0 (5.9)	11.3 (3.9)	1.7 (4.6)
Combination Treatment	13.0 (5.8)	11.1 (3.9)	2.0 (4.4)

## Discussion

Liver transplant in patients within Milan criteria [13] is based on well-established data and excellent evidence of outcome [14], however, there are data from other studies suggesting that transplants in those exceeding Milan criteria do not adversely impact survival [15-18]. Currently, transplant centers utilize LRT to prevent listed patients from dropping out due to tumor progression [5]. Despite the increased use of LRT in recent years, its benefit in reducing mortality and recurrence of HCC following liver transplant has been inconsistently reported in the existing literature, and the overall scope of evidence supporting this practice is limited [6-12]. Novel surgical techniques used in HCC with portal vein thrombosis have shown an evolving role from advanced hepatobiliary centers [19].

Considering data limitation in mind, the results of this study suggest LRT has no statistically significant difference in patient survival and tumor recurrence in managing HCC patients awaiting transplant with no therapy. While not statistically significant, there were trends toward improvements in five-year post-transplant survival and recurrence-free survival when combination LRT was utilized, despite that typically combined LRT therapy is used for larger and potentially more aggressive HCCs. These findings bear significant clinical relevance in emphasizing the safety of combination LRT in the management of HCC patients awaiting transplant. This is particularly important when considering the revisions made to the OPTN liver allocation policy recently affecting the maximum number of exception points granted to HCC patients awaiting transplant in addition to mandating a six-month wait period before points may be granted [14,20]. 

Although limited by a retrospective design, this study is, to our knowledge, the largest retrospective study published in the U.S. comparing those HCC patients receiving no LRT with those receiving different types and combinations of therapies. At this time, it would be logistically challenging to perform a prospective study of similar scope, particularly when considering the ethical question posed by randomizing patients to a control arm in which the mandatory six-month wait period for the exception would present a large window for tumor progression and subsequent loss of transplant candidacy. Although, a large-scale randomized controlled trial will be more appropriate to determine a systematic approach to the selection and implementation of different LRT modalities while ensuring maximal patient response and long-term safety. Performing such trials bear other complexities such as cost of such trial, presence of regional expertise with different LRTs, and feasibility of the treatment options. 

There are additional limitations to this study which are inherently posed by the database utilized. Given that the completeness of OPTN data is reliant upon a manual population of all patient data fields, it is possible that inconsistencies in the clinical and demographic parameters reported for patients exist. For example, the HCC recurrence rate observed in this study is lower than anticipated, with this finding, likely attributed to the underreporting of HCC recurrence to the OPTN. Additionally, the choice to use a Medicare database limits the generalizability of our findings, as patients with private insurance are not accounted for and thus could experience different outcomes. Finally, as the database utilized does not include patients who did not receive liver transplantation, neither the drop-out rate for this cohort nor the potential effect of LRT in reducing the dropout rate can be analyzed.

It is worth noting that the data collection period of this study, spanning 2003-2013, represents a time of significant development in both imaging criteria for the diagnosis and staging of HCC as well as the use of MELD scores in the prioritization of patients following the initial institution of the OPTN allocation policy. As such, it is possible that the impact of these advancements on the effects of bridging therapy is not fully appreciable in the results presented here. This study has limited applicability to the current paradigm due to the current six-month waiting period which was not in effect at the time of the study. Currently, no treatment for six months would not be ethically justified for patients who are candidates for LRT. As previously stated, a prospective study is required to determine a systematic approach to the use of LRT in managing this patient population, especially in the context of the mandatory wait period instituted in 2015, and the OPTN staging protocol adopted in 2016 [14], advancements which are not accounted for in this study. Furthermore, a prospective analysis of a contemporary cohort would allow a more thorough investigation of explant pathology given the limited availability of this data to those patients transplanted after 2012.

## Conclusions

Different types and combinations of LRT were used to prevent tumor growth and progression of disease beyond the Milan criteria to achieve transplant for patients listed based on a primary diagnosis of HCC. Our study is the largest retrospective study comparing liver transplant patients with HCC who receive LRT to none for outcomes of survival benefit and tumor recurrence. Though our study did not show any statistically significant benefit of single or combination LRT, the outcomes encourage the safe and feasible use of LRT as a bridging therapy. Larger multi-center prospective studies will be required to achieve the effect size, however practically not feasible. This study outcome definitely encourages further examination of evidence from other sources to help with synthesizing further guidelines for LRT. At this stage, LRT use and selection should be decided based on transplant center expertise.

## Appendices

Appendix 1

**Table 10:** Recipient and donor baseline characteristics by treatment (n=4,669) MELD - Model for End Stage Liver Disease TX - Transplant LD - Living Donor DCD - Donation after Circulatory Death NON DCD - Not Donation after Circulatory Death DRI - Disease Risk Index

Characteristics	No Treatment (n=2,257)	Single Therapy (n=1,790)	Combination Therapy (n=6,22)	p-value
	%	%	%	
Recipient Variables				
Age				< 0.001
19-35	0.3	0.3	0.3	
36-40	0.3	0.1	0.3	
41-55	19.4	17.0	15.8	
56-70	73.6	73.6	71.9	
>=71	6.4	9.1	11.7	
Gender				0.223
Male	73.8	71.3	72.4	
Female	26.2	28.7	27.7	
Race				0.142
White	64.6	64.6	64.2	
Black	9.6	9.3	8.0	
Hispanic	17.9	16.4	16.6	
Other	8.0	9.7	11.3	
BMI				0.093
10-18.5	0.8	1.6	0.6	
18.5-25	24.5	26.8	27.2	
25-30	40.1	37.3	39.6	
>30	34.6	34.4	32.6	
Missing	0.0	0.1	0.0	
Blood Type				0.065
A	37.9	36.8	35.5	
B	11.7	14.2	16.4	
AB	3.7	3.6	4.0	
O	46.7	45.5	44.1	
Calculated MELD				0.035
<10	27.9	31.4	29.9	
10-14	37.9	38.9	38.4	
15-19	18.9	17.0	19.1	
20-24	8.4	7.0	7.4	
25-29	2.3	2.3	1.3	
30-34	2.0	12	1.1	
35-39	1.0	0.7	0.8	
>=40	0.9	0.4	0.3	
Unknown	0.7	1.0	1.6	
College Degree	39.1	40.0	37.5	0.529
Employed	8.4	7.9	7.7	0.791
Diabetes	35.2	34.2	32.6	0.460
Peripheral Vascular Disease	1.0	1.4	1.3	0.450
Partial/split liver	2.3	1.8	3.9	0.015
Liver and Kidney TX	3.8	2.7	2.4	0.089
Primary Insurance Types				< 0.001
Public	63.9	78.3	80.2	
Private	32.7	17.3	16.2	
Other	3.3	4.4	3.5	
Donor Variables				
Donor Age				0.151
0-18	7.4	8.5	8.4	
19-35	29.4	27.3	28.6	
36-50	26.2	25.5	29.6	
>=51	36.9	38.7	33.4	
Donor Race				0.607
White	64.4	65.9	66.1	
Black	16.0	16.5	17.4	
Hispanic	15.0	13.5	12.4	
Other	4.5	4.1	4.2	
Donor Diabetes	13.1	11.8	12.1	0.423
Donor Types				0.006
LD	0.9	0.5	1.9	
DCD	5.5	5.4	6.9	
NON-DCD	93.6	94.2	91.2	
Organ Share Type				0.037
Local	78.0	81.3	81.8	
Region	18.5	16.0	14.6	
Nation	3.5	2.7	3.5	
DRI				0.451
0.0<=DRI<1.0	0.2	0.1	0.0	
1.0<=DRI<1.5	30.5	30.5	32.0	
1.5<=DRI<2.0	35.0	34.6	30.6	
2.0<=DRI<2.5	19.2	21.0	22.4	
DRI>=2.5	6.7	6.8	6.9	
Unknown	8.4	7.1	8.2	
Cold Ischemic Time (Hours)				0.352
0-6	56.1	56.9	56.9	
7-12	38.4	38.3	36.3	
>=13	1.8	2.1	2.9	
Missing	3.7	2.7	3.9	

Appendix 2

**Table 11:** Pre-transplant tumor variables (n = 4,669)

Variable	No Treatment	Single Therapy	Combination Therapy	p-value
	%	%	%	
Pre-transplant tumor number				0.897
1-2	92.3	92.6	92.3	
>=3	7.7	7.4	7.7	
Pre-transplant tumor size (CM)				0.001
< 2	17.2	13.4	15.3	
2 - 5	76.7	79.9	74.8	
6 – 8	5.7	6.4	9.3	
> 8	0.4	0.3	0.6	

Appendix 3

**Table 12:** Recipient explant pathology by treatment (n=1,674)

Pathology	No Treatment	Single Therapy	Combination Therapy	p-value
	%	%	%	
Tumor number				< 0.0001
1	22.2	15.3	11.4	
2	8.8	6.1	6.3	
3	4.4	4.2	3.7	
4	2.4	1.9	1.1	
5	1.3	1.1	1.0	
>5	1.8	1.3	1.8	
Infiltrative	0.2	0.3	0.0	
Unknown	58.9	69.9	74.8	
Tumor size (CM)				< 0.0001
< 8.5 CM	37.8	27.7	23.0	
>= 8.5 CM	3.2	2.1	2.3	
Unknown	59.1	70.2	74.8	
Tumor differentiation				< 0.0001
Complete tumor necrosis	7.7	4.9	4.2	
Poor	2.8	2.7	2.1	
Moderate	20.5	15.3	12.7	
Well	10.1	7.3	6.3	
Unknown	59.0	69.9	74.8	
Vascular invasion				< 0.0001
None	34.7	25.0	20.6	
Microvascular	5.8	4.6	3.7	
Macrovascular	0.7	0.5	1.0	
Unknown	58.9	69.9	74.8	
% Tumor necrosis, mean (SD)	0.3 (0.4)	0.3 (0.4)	0.3 (0.4)	0.992
